# Mesothelioma patient derived tumor xenografts with defined *BAP1* mutations that mimic the molecular characteristics of human malignant mesothelioma

**DOI:** 10.1186/s12885-015-1362-2

**Published:** 2015-05-08

**Authors:** Neetu Kalra, Jingli Zhang, Anish Thomas, Liqiang Xi, Mitchell Cheung, Jacqueline Talarchek, Sandra Burkett, Maria G Tsokos, Yuanbin Chen, Mark Raffeld, Markku Miettinen, Ira Pastan, Joseph R Testa, Raffit Hassan

**Affiliations:** 1 Thoracic and GI Oncology Branch, Center for Cancer Research, National Cancer Institute, National Institutes of Health, Bethesda, MD 20892 USA; 2 Laboratory of Pathology, Center for Cancer Research, National Cancer Institute, National Institutes of Health, Bethesda, MD 20892 USA; 3 Cancer Biology Program, Fox Chase Cancer Center, Philadelphia, PA 19111 USA; 4 Molecular Cytogenetics Core Facility, National Cancer Institute, Frederick, MD 21702 USA; 5 Beth Israel Deaconess Medical Center, Harvard Medical School, Boston, MA 02215 USA; 6 Laboratory of Molecular Biology, Center for Cancer Research, National Cancer Institute, National Institutes of Health, Bethesda, MD 20892 USA

**Keywords:** Mesothelioma, Malignant effusions, *BAP1*, *CDKN2A*, Patient derived tumor xenografts

## Abstract

**Background:**

The development and evaluation of new therapeutic approaches for malignant mesothelioma has been sparse due, in part, to lack of suitable tumor models.

**Methods:**

We established primary mesothelioma cultures from pleural and ascitic fluids of five patients with advanced mesothelioma. Electron microscopy and immunohistochemistry (IHC) confirmed their mesothelial origin. Patient derived xenografts were generated by injecting the cells in nude or SCID mice, and malignant potential of the cells was analyzed by soft agar colony assay. Molecular profiles of the primary patient tumors, early passage cell cultures, and patient derived xenografts were assessed using mutational analysis, fluorescence *in situ* hybridization (FISH) analysis and IHC.

**Results:**

Primary cultures from all five tumors exhibited morphologic and IHC features consistent to those of mesothelioma cells. Mutations of *BAP1* and *CDKN2A* were each detected in four tumors. *BAP1* mutation was associated with the lack of expression of *BAP1* protein. Three cell cultures, all of which were derived from *BAP1* mutant primary tumors, exhibited anchorage independent growth and also formed tumors in mice, suggesting that BAP1 loss may enhance tumor growth *in vivo*. Both early passage cell cultures and mouse xenograft tumors harbored *BAP1* mutations and *CDKN2A* deletions identical to those found in the corresponding primary patient tumors.

**Conclusions:**

The mesothelioma patient derived tumor xenografts with mutational alterations that mimic those observed in patient tumors which we established can be used for preclinical development of novel drug regimens and for studying the functional aspects of *BAP1* biology in mesothelioma.

**Electronic supplementary material:**

The online version of this article (doi:10.1186/s12885-015-1362-2) contains supplementary material, which is available to authorized users.

## Background

Malignant mesothelioma is an asbestos-related aggressive tumor with poor prognosis, occurring in the mesothelial lining of pleural or peritoneal cavities. Malignant mesothelioma is a deadly and clinically challenging disease due to its low incidence, resistance to most chemotherapies, and complexity of tumor anatomy. Despite multimodality therapy, the median overall survival is less than a year for patients with pleural tumors, and the 5-year survival rate is less than 15% for peritoneal mesothelioma [1].

The development and evaluation of new therapeutic approaches for malignant mesothelioma has been sparse due, in part, to lack of suitable tumor models. Efforts to generate clinically relevant tumor models have focused on two main approaches in the recent years: generation of primary tumor-derived cell lines and of mouse models. Primary tumor-derived cell lines have adapted to growth outside a natural tumor microenvironment and due to selective processes associated with long term culturing will develop genetic changes that are distinct from the genetic stress imposed on tumors in patients [2-4]. Therefore even when propagated *in vivo*, they may not be suitable for preclinical testing of anti-cancer drugs or for studying the underlying gene expression associated with novel drug responses. Mouse models generated *via* engraftment of primary human tumors into immune-compromised mouse models have become increasingly popular for preclinical testing of anticancer drugs. However their usefulness depends upon the preservation of biological and morphological characteristics of the primary tumors [5]. Many of the currently available mesothelioma cell lines do not form tumors in mice, and others have been propagated in culture for many passages, leading to various cytogenetic changes. Thus, these lines often do not show much similarity with the original tumors [6].

The most common genetic alterations associated with mesothelioma, including C*DKN2A* deletions and *NF2* mutations, have been known for about two decades [7-9]. More recently, mutations in the *BAP1* tumor suppressor gene have been observed in 20-25% of mesothelioma tumor samples [10,11]. BAP1, a nuclear ubiquitin hydrolase, plays an important role in various cellular processes including cell proliferation, DNA repair and regulation of gene expression at the chromatin level [12].

This study describes molecular and immunohistochemical characterization of five primary mesothelioma cell lines. By comparing mutational and immunohistochemical profiles between primary cell cultures, and patient derived xenografts, we report the stability of both the genetic profile and protein expression in the xenografts, highlighting their potential for exploring genetic changes associated with responses to established and novel drugs.

## Methods

### Pathological examination of the original tumor specimens

All patients whose samples were utilized for this study were enrolled in Institutional Review Board approved protocols at the Center for Cancer Research, National Cancer Institute. All patients provided written informed consent which allowed the storage and use of body fluids, tumor samples and data that were collected for future research. Tumor samples obtained from five patients at the time of diagnosis or at the time of debulking surgery were evaluated by a pathologist to establish the diagnosis and characterize the subtype of mesothelioma.

### Establishment of early-passage mesothelioma cell cultures

Early passage primary mesothelioma cell cultures were isolated from ascites or pleural fluid obtained from mesothelioma patients at the National Cancer Institute. The ascites or pleural fluid (100–1000 mL) was centrifuged at 1000 rpm at room temperature for 3 minutes; the cell pellets were washed twice with phosphate buffered saline (PBS), and red blood cells were removed using a BD Pharm Lyse™-Lysing Buffer kit (BD Bioscience, NJ), according to the manufacturer’s instructions, and washed again two times with PBS. The cells were then resuspended in RPMI 1640 (Invitrogen, CA) supplemented with 2 mM glutamine, 100 units penicillin-streptomycin, and 1 mM sodium pyruvate (each from Invitrogen, CA) plus 20% fetal bovine serum (FBS) (Lonza, MD). The cells were seeded into 175 mL culture flasks at a density of 2.5-4.0 × 10^5^ cells/ml. After incubating at 37°C in a humidified, 5% CO_2_ atmosphere overnight, the medium containing non-adherent cells was replaced with fresh medium. The cultures were maintained by changing the medium depending upon the growth of the cells. To authenticate these cell lines for future use by us or other investigators we performed Short Tandem Repeats (STR) analysis of these cells.

### Immunohistochemistry

Cells were detached using trypsin-EDTA and then washed and centrifuged. The cell pellets were fixed in formalin and embedded in paraffin. Tumor sections were prepared, and immunohistochemical studies were carried out for the mesothelial markers calretinin, WT1, CK5/6, and mesothelin and BAP1 using specific antibodies (Santa Cruz Biotechnology, TX). All immunostaining was carried out using an automated Ventana system (Ventana Medical Systems, AZ) using their UltraView polymer based detection system. IHC staining was scored semiquantitatively as follows: negative (less than 5% of cells stained), 1+ positive (5- 50%), and 2+ positive (50-100%).

### Electron microscopy

Cells from all five cell lines were washed in PBS, fixed in PBS-buffered 2.5% glutaraldehyde (Sigma Chemicals, MO), postfixed in 0.5% osmium tetroxide and embedded into Spurr’s epoxy resin (Ladd Research Industries, VT). Ultrathin sections were stained with uranyl acetate-lead citrate and viewed in a Phillips CM10 transmission electron microscope.

### Spectral karyotyping and fluorescent in situ hybridization

Spectral karyotyping was performed according to the manufacturer’s protocol using 24-color human SKY paint probes (Applied Spectral Imaging, CA). Fluorescent *in Situ* Hybridization (FISH) analysis was carried out using a *CDKN2A* probe (Abbott Molecular, IL) encompassing the overlapping genes encoding p16^INK4a^ and p14^ARF^. Spectral images of the hybridized metaphases were acquired using a SD301 SpectraCubeTM system (Applied Spectral Imaging Inc., CA) mounted on top of an epi-fluorescence microscope Axioplan 2 (Zeiss). Images were analyzed using Spectral Imaging 6.0 acquisition software (Applied Spectral Imaging Inc., CA). At least 10 SKY hybridized metaphases were analyzed in this experiment. FISH analyses were performed under an Axioplan 2 (Zeiss) fluorescence microscope coupled with a CCD camera (ASI) and images were captured with FISH view 5.5 software (Applied Spectral Imaging Inc., Vista, CA).

### Mutation analysis

Genomic DNA was extracted from early passage cells using a DNA isolation kit (Mo-Bio Laboratories, CA). PCR was done using exon specific primers for the entire coding region of *BAP1*, the tyrosine kinase domain of the *EGFR*, [13] and exons 2–9 of *TP53*. Polymerase Chain Reaction (PCR) products were purified and subjected to Sanger sequencing on an ABI PRISM sequencing apparatus (ABI Prism 310 Genetic Analyser, Applied Biosystems, NY). In addition, a panel of hotspot mutations in 7 genes (*AKT1*, *BRAF*, *EBRR2*, *EGFR*, *KRAS*, *NRAS*, and *PIK3CA*) was analyzed with pyrosequencing for single-nucleotide variants (SNVs) or with fragment analysis for insertions/deletions (indels). Briefly PCR amplification was initially performed with primers flanking the mutation hotspot under co-amplification at lower denaturation temperature (COLD)-PCR conditions and was followed by targeted pyrosequencing on a PyroMark Q24 instrument (Qiagen) or by capillary electrophoresis using a Genetic Analyzer 3130xl (Life Technologies) [14-20].

### Flow cytometry

Mesothelin expression on early and late passage primary cell cultures were evaluated by flow cytometry using the anti-mesothelin primary antibody (MN) as previously described [21].

### Western blotting

Monolayers of confluent cells were washed twice in PBS and then lysed in 1× Cell Lysis Buffer supplemented with 1 mM phenylmethylsulfonylfluoride (Cell Signaling Technology, MA). Fifty micrograms of total protein were subjected to SDS-polyacrylamide gel electrophoresis (Invitrogen, CA) for each cell line followed by immunoblotting with mouse monoclonal E-cadherin, N-cadherin and vimentin antibodies (BD Bioscience, NJ) and BAP1 antibody (Santa Cruz Biotechnology, CA) (1:1,000 in 5% blocking reagent in Tris-buffered saline/Tween-20) overnight at 4°C. The following day, blots were incubated with goat anti-mouse IgG conjugated with horseradish peroxidase (Santa Cruz Biotechnology; 1:1,000 dilution) for 1 hour at 25°C. Signals were visualized with enhanced chemiluminescence reagent (Amersham Pharmacia Biotech, NJ) on X-ray film (Eastman Kodak, NY).

### Soft agar colony assay

Twenty thousand cells were suspended in 0.3% low melting agarose (Lonza, Rockland, ME) in RPMI containing 20% FBS. This suspension was overlaid onto a solid layer of 0.6% agarose in a 6-well plate. The cells were treated with fresh RPMI containing 20% FBS every other day. After 2–3 weeks, depending upon the growth, the cells were fixed with methanol, stained with 0.02% crystal violet, and photographed at ×10 magnification.

### *In vivo* xenograft studies

All animal experiments were performed in accordance with NIH guidelines and approved by the NCI Animal Care and Use Committee. Cultured cells (5×10^6^-10×10^6^ cells) were injected subcutaneously into the dorsal side of three nude or SCID mice. The animals were examined every week for the development of tumors. All animal care was done in accordance with institutional guidelines. The single tumor for each primary cell was excised and fixed in 10% formalin to process for routine histopathological examination and to perform immunohistochemical staining for the earlier mentioned markers.

## Results

### Primary mesothelioma cultures established from malignant effusions of patients with mesothelioma

Table 1 summarizes characteristics, diagnosis and course of treatment of patients with malignant mesothelioma from whom the primary cultures were derived. Four of the five tumors had epithelial histology and one was predominantly epithelial with focal sarcomatous features. All tumors were positive (15-100% of tumor cells) for mesothelioma-related markers mesothelin and calretinin by IHC. Four of the tumors were also positive for the cytokeratin 5/6.

**Table 1:** **Clinical and pathological characteristics of patients from which primary cultures were established**

Cell lines	Sex	Age (dx)	Ethnicity	Diagnosis	Patient tumor		TNM stage	Treatment	Survival from diagnosis
					Histology	Positive markers			
NCI-Meso16	M	72	Caucasian	Pleural	Epithelioid	Mesothelin: 2+, 30%, Calretinin, WT1, CK5/6(focal)	cT4N1M0 (IV)	Cisplatin + Pemetrexed (6 cycles) with SS1P (first two cycles)	8 months
NCI-Meso17	F	63	Caucasian	Peritoneal	Predominantly epitheliod with focal sarcomatous features	Mesothelin: 2+, 15%, WT1, CK5/6, CA125	NA	Debulking + IP Cisplatin + doxorubicin	8 years
								IP Pemetrexed + IV Cisplatin	
								IP Taxol	34 months
								Debulking + IP mitomycin + doxorubicin	
								phase II investigationsl agent	
NCI-Meso18	M	60	Caucasian	Pleural	Epithelioid	Mesothelin: 3+, 100%, Calretinin, CK5/6, WT1	cT3N0M0 (III)	Cisplatin + Pemetrexed	
								EPP	
								Cisplatin + Pemetrexed	
								Gemcitabine	
								Navelbine	
								phase II investigational agent	
NCI-Meso19	M	19	African	Pleural	Epithelioid	Mesothelin: 2+, 100%, Calretinin	T4N3MX (IV)	Died before any specific treatment	3 months
NCI-Meso21	M	66	Caucasian	Pleural	Epithelioid	Mesothelin: 3+, 75%, WT1, calretinin, CK5/6	T4 (IV)	Cisplatin + Pemetrexed (6 cycles) with SS1P (first two cycles)	13 months

NA, not applicable; IP, intraperitoneal; IV, intravenous; EPP, extra-pleural pneumonectomy. Patient tumor mesothelin expression is reported based on the intensity of staining (1+ to 3+) and percentage of cells stained (1-100%).

### Morphologic features, electron microscopy and immunohistochemical staining of primary cell cultures are indicative of their being of mesothelial origin

Primary cultures from all five tumors exhibited morphologic and immunohistochemical features consistent to those of mesothelial cells. All primary cultures except NCI-Meso17 grew as adherent monolayers characterized by polygonal epithelial-type cells that tended to group together as clusters in a colony-like formation. In addition to polygonal epithelial cells, NCI-Meso17 cells also included spindle like cells, which became the predominant cell population as the cells were maintained in culture.

**Figure 1:**
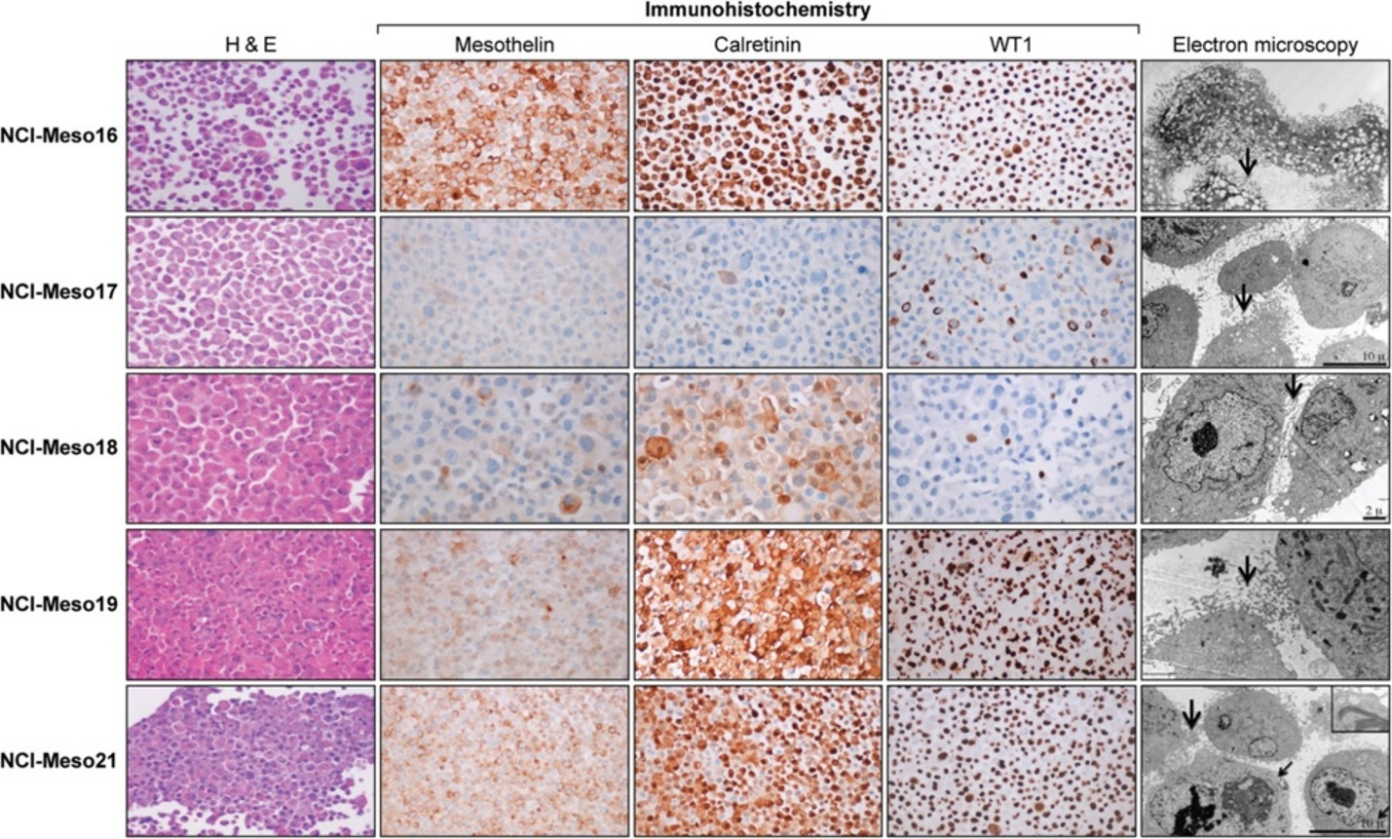
Characteristics of the primary mesothelioma cell cultures. **Left panel**: Hematoxylin and eosin staining of primary cell cultures. **Middle panels**: Representative immunohistochemical stains of mesothelioma cell culture pellets that were formalin-fixed and paraffin-embedded. NCI-Meso16, NCI-Meso19 and NCI-Meso21 cells were strongly and diffusely positive for mesothelin, calretinin and WT1. NCI-Meso18 cells expressed primarily calretinin in a smaller percentage of cells, whereas NCI-Meso17 cells showed a small percentage of WT-1 positive cells. **Right panel**: Electron microscopic images of primary tumor cells which exhibit numerous long and complex surface villi (thick arrows) that are characteristic of mesothelioma cells. The thin arrow in the electron microscopic image of the NCI-Meso21 cells points to condensed aggregates of intermediate filaments indicative of tonofilamens, shown in detail at a higher magnification in the inset.

Cell blocks were prepared from early-passage (≤5 passages) mesothelioma cells and sections were evaluated by hematoxylin and eosin (H&E) and IHC (Figure 1 and Table 1). IHC revealed high levels of expression of mesothelin (2+ to 3+), and WT1 and calretinin (3+) in three samples (NCI-Meso16, NCI-Meso19, NCI-Meso21). NCI-Meso17, which was derived from a patient who had biphasic disease, was negative for most of the above markers, but retained a few WT-1 positive cells. NCI-Meso18, which was derived from an epithelioid mesothelioma, retained moderate expression of calretinin and minimal expression of mesothelin and WT1.

Electron microscopy analysis further supported the mesothelial origin of these cells, as the long irregular and branched microvilli characteristic of mesothelioma cells were present in all the cell cultures (Figure 1). We also noticed the presence of intracytoplasmic intermediate filaments in all the primary cells and glycogen bodies in some of them. Tonofilaments could also be seen in one of the cell cultures (NCI-Meso21). Thus, the electron microscopy data strongly indicated that the primary cell cultures are of mesothelioma origin.

The cell morphology was retained throughout the 30 passages we tested, and we performed STR analysis at different passages in order to eliminate the possibility of cross contamination among cell cultures (Additional file 1). STR analysis showed only modest changes in DNA fingerprinting profiles between primary tumors and the corresponding cell cultures [22]. We also assessed cell surface expression of mesothelin by flow cytometry in early and late passage cells (Additional file 2). Primary cells which expressed mesothelin at early passages (NCI-Meso16, NCI-Meso18, NCI-Meso19, and NCI-Meso21) continued to express mesothelin in late passage cultures. NCI-Meso17 which did not express mesothelin in early passages remained mesothelin negative in late passages. Following thawing, cryopreserved cells could be propagated in culture without a noticeable change in growth and morphology. In all subsequent experiments, cells were used between the third to sixth passages in culture.

### Cytogenetic analysis reveals abnormal karyotype in all five primary cultures

Spectral karyotyping of early passage cell cultures uncovered multiple structural and numerical chromosome abnormalities in all five cultures as summarized in Additional file 3. The more frequent chromosomal rearrangements included clonal rearrangement or deletions of chromosomes 1 (4/5 cell cultures), 3 (3/5), 9 (4/5) and 22 (2/5). The karyotype analysis showed a very high number of structural abnormalities in NCI-Meso19, a cell culture derived from a mesothelioma of a young patient harboring a somatic *TP53* mutation. These results are consistent with earlier reports showing frequent alterations of 1p, 3p, 9p, and 22 in human mesothelioma specimens and derived cell lines [23]. To assess the stability of karyotypic changes, we evaluated karyotypes of early and late passage cells. This revealed only minor karyotypic changes between passages which are summarized in Additional file 3.

### Epithelial-mesothelial markers are present in all five primary cell cultures

All five cell cultures expressed N-cadherin, consistent with their mesothelial origin as described previously [3]. E-cadherin, an epithelial marker, was also expressed in all cultures except NCI-Meso17, which was derived from the patient who had biphasic disease. NCI-Meso17 had high expression of the mesenchymal marker, vimentin (Additional file 4).

### Frequent BAP1 mutations in primary cell cultures

**Figure 2:**
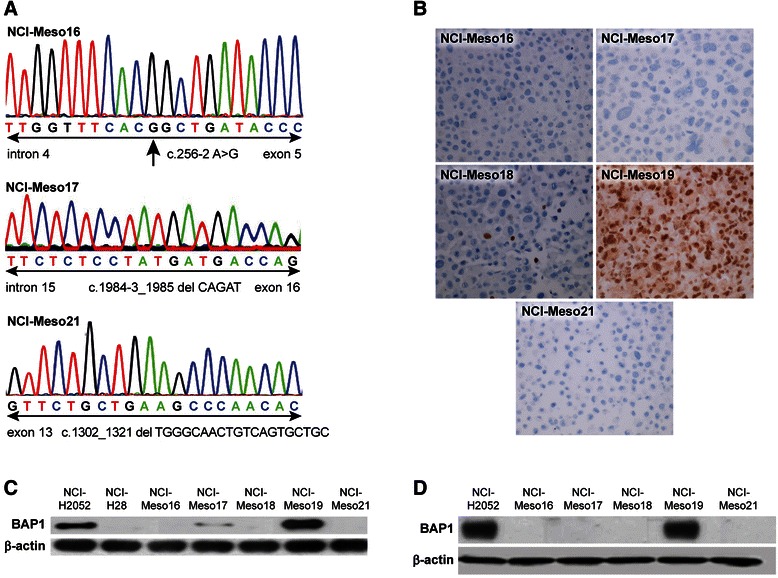
*BAP1* alterations in primary mesothelioma cells. Chromatograms showing *BAP1* mutations **(A)**. Arrow in the top panel indicates the location of mutation. NCI-Meso16 cells had a splice site mutation in intron 4 of *BAP1*. NCI-Meso17 and NCI-Meso21 had frame shift mutations at the intron15/exon16 junction and in exon 13 of *BAP1* gene, respectively. Immunohistochemistry **(B)** showing lack of expression of BAP1 protein in cells with mutant *BAP1*. Only NCI-Meso19 cells which were *BAP1* wild-type expressed the BAP1 protein. Western blot **(C)** showing the expression of BAP1 in primary mesothelioma cells. For western blot, mesothelioma cell lines NCI-H28 and NCI-H2052 were used as controls for mutant and wild-type *BAP1* respectively. A faint BAP1 band was observed for NCI-Meso17 cells, which exhibits a 5-bp deletion in exon 13. Western blot showing **(D)** the expression of BAP1 in late passage primary mesothelioma cells. The faint BAP1 band which was observed for NCI-Meso17 cells in early passage was absent in late passage.

Mutation analysis of early passage cells revealed *BAP1* mutations in 4 of 5 cell cultures (Figure 2A and Table 2). NCI-Meso16 cells had a splice site mutation in intron 4 of *BAP1*. NCI-Meso17 and NCI-Meso21 had frame shift mutations at the intron15/exon16 junction and in exon 13 of *BAP1* gene, respectively (Table 2 and Figure 2A). NCI-Meso18 had a large *BAP1* deletion. We found a clear correlation between *BAP1* mutation and the absence of BAP1 protein by IHC (Figure 2B). For western blot analysis, we used the mesothelioma cell lines NCI-H28 (harboring a *BAP1* nonsense mutation) and NCI-H2052 (wild type for *BAP1*) as positive and negative controls, respectively, for the presence of a BAP1 protein (Figure 2C). BAP1 protein expression detected by western blot was preserved after serial passages with late passage cells showing similar expression patterns as early passage cells (Figure 2D). NCI-Meso17 cells which exhibits a 5-bp deletion in exon 13 demonstrated a faint BAP1 band in early passages which was not detected in late passage cells.

**Table 2:** **Mutations in early passage malignant mesothelioma primary cell cultures**

Cell lines	*CDKN2A*deletion	*BAP1*	*NF2*	*TP53*	*EGFR*	*KRAS*	*NRAS*	*BRAF*	*PIK3 CA*	*ERBB2*	*AKT1*
NCI-Meso16	wt	Intron 4 spice site mutation c.256-2A > G, Hm	Wt	Wt	Wt	Wt	Wt	Wt	Wt	Wt	Wt
NCI-Meso17	Hm	Deletion of CAGAT at intron 15/exon 16 junction. c.	Wt	Wt	Wt	Wt	Wt	Wt	Wt	Wt	Wt
		1984-3_1985delCAGAT									
NCI-Meso18	Hm	Large deletion	Wt	Wt	Wt	Wt	Wt	Wt	Wt	Wt	Wt
NCI-Meso19	Hm	Wt	Large deletion	*P322S	Wt	Wt	Wt	Wt	Wt	Wt	Wt
NCI-Meso21	Hz	Homozygous 20 bp deletion in exon 13. c.	Wt	Wt	Wt	Wt	Wt	Wt	Wt	Wt	Wt
		1302_1321delTGGGCAA CTGTCAGTGCTGC									

All 5 cell lines were analyzed for mutations in *BAP1* whole gene, *P53* exons 2–9, *EGFR* Exons 18–24, *KRAS* and *NRAS* Codon 12, 13 and 61, *BRAF*-599-601, *PIK3CA* Exon 9 and 20, *ERBB2* insertion in Exon 20 and *AKT1* codon 17. *TP53 heterozygous mis-sense mutation in codon 322 (P322S, Exon 9), polymorphism codon 72 (P72R, Exon 4). Hm, homozygous. Hz, heterozygous. Wt, wild-type.

### Frequent deletions of CDKN2A in primary cell cultures

We screened cell cultures for deletions of the *CDKN2A* locus in chromosome 9p21 by FISH analysis on primary cell cultures. Four of the samples were found to have homozygous deletions of *CDKN2A* (Table 2), consistent with rates reported in malignant mesothelioma cells [7,24-27]. The percentage of nuclei with a homozygous deletion pattern was more than 60% in four cultures. One culture had primarily wild type cells, with less than 10% of cells showing a homozygous deletion of *CDKN2A*.

### Mutations in other genes tested

We also performed sequence analysis of *NF2* and *TP53*, and hot spot mutation screening for several oncogenes, as summarized in Table 2. A large deletion in *NF2* and a missense mutation in exon 8 (P322S) of the *TP53* gene were found in one of the primary cultures (Table 2). We did not see mutations in any of the other genes tested.

### *In vitro* soft agar colony growth of mesothelioma cells correlates with tumorigenic potential *in vivo*

**Figure 3:**
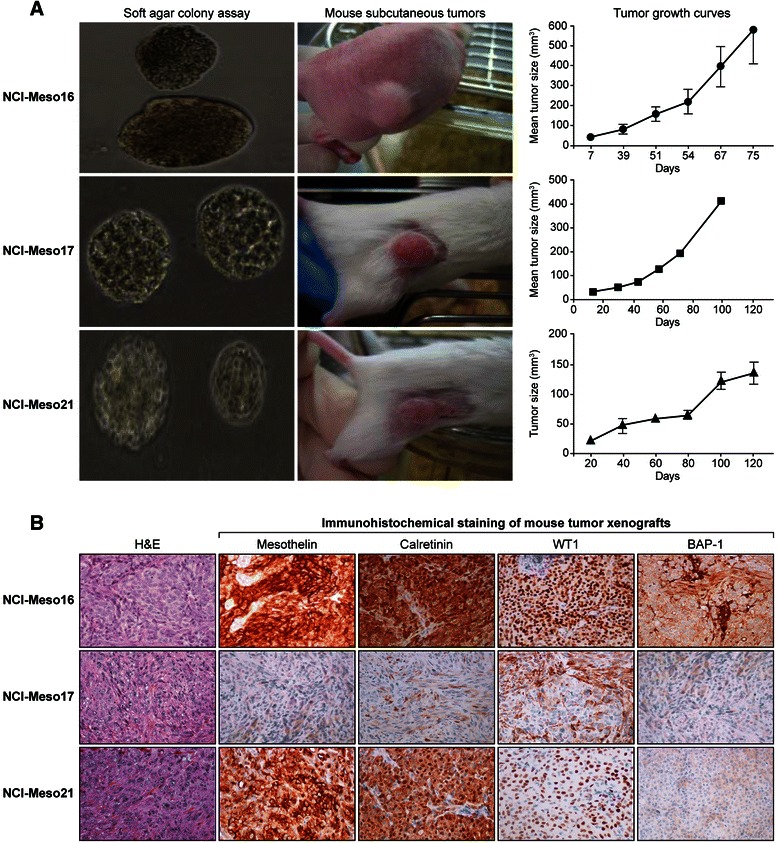
Colony-forming ability and tumorigenicity of primary mesothelioma cells. Colony formation *in vitro* in 0.3% soft agar **(A left panel)**. Colonies could be seen for NCI-Meso16, NCI-Meso17 and NCI-Meso21 cell cultures, each of which showed *BAP1* mutations. Tumorigenic potential of these cell cultures was also investigated by subcutaneous injection of cells with matrigel in nude or SCID mice. Representative photographs of subcutaneous tumors derived following injection of NCI-Meso16, NCI-Meso17 and NCI-Meso21 cells is shown **(A middle panel)** along with the corresponding tumor growth curves **(A right panel)**. Immunohistochemical staining for different markers showed the same pattern in tumor xenografts as in the matching primary cell cultures **(B)**.

A soft agar colony assay was performed to examine anchorage independent growth. Three of the five cultures (NCI-Meso16, NCI-Meso17 and NCI-Meso21) formed colonies within 3 weeks (Figure 3A). Cells that formed colonies in the *in vitro* assay also formed visible tumors in mice within 3–8 weeks. Notably, all three of these cell cultures had *BAP1* mutations. Tumor growth varied among the cell cultures, with a tumor volume of 100 mm^3^ achieved between 40 to 80 days after inoculation (Figure 3A).

### Early passage mesothelioma cells and their tumor xenografts retain molecular features of human malignant mesothelioma

The expression of mesothelioma specific immunohistochemical markers in primary cell cultures were preserved in the tumor sections from mouse xenografts (Figure 3B). Furthermore, the xenografts lacked BAP1 expression, as shown by the absence of nuclear staining, similar to the corresponding primary cells. This finding was further supported by detection of the identical *BAP1* mutations in tumor xenografts, and the corresponding primary tumor samples and primary cell cultures (Table 3). In addition, xenograft tumors exhibited the same *CDKN2A* deletion status as in primary cell cultures, except in one case where most but not all cells had a homozygous deletion of *CDKN2A*, whereas the reverse was true in the corresponding primary cell culture. This suggests that the tumor cells with loss of *CDKN2A* had a selective growth advantage *in vivo*. The two remaining tumor samples completely matched with their primary cultures for their *CDKN2A* deletion status, further confirming that primary cells obtained from patient’s malignant effusions have the same genetic alterations as in the corresponding primary human tumor.

**Table 3:** **Comparison of patient tumors with primary cell culture and with patient derived xenograft in terms of** ***BAP1*** **mutation and** ***CDKN2A*** **deletion**

*BAP1*mutation				*CDKN2A*deletion by FISH			
	Patient tumor	Primary cell culture	Mouse xenograft		Patient tumor	Primary cell culture	Mouse xenograft
NCI-Meso16 (splice site mutation in Intron 4)	Present	Present	Present	NCI-Meso16 (wt)	Homozygous deleted cells some wt cells	Mostly wt. Some homozygous deleted cells	Mostly wt. Some homozygous deleted cells
NCI-Meso17 (5 bp deletion in intron 15- Exon 16 junction)	Present	Present	Present	NCI-Meso17 (Homozygous deletion)	Present	Present	Present
NCI-Meso18 (large deletion)	Sample not available	large deletion	Does not form tumor	NCI-Meso18 (Homozygous deletion)	Sample not available	Present	Does not form tumor
NCI-Meso19 (Wt)	Wt	Wt	Does not form tumor	NCI-Meso19 (Homozygous deletion)	Present	Present	Does not form tumor
NCI-Meso21 (20 bp deletion in Exon 13)	Sample not available	Present	Present	NCI-Meso21 (Heterozygous deletion)	Sample not available	Present	Present

Wt- wild-type.

## Discussion

There is a need to develop better preclinical tumor models to evaluate new therapeutic approaches for mesothelioma. Patient derived tumor xenograft models are increasingly being recognized as a robust approach for evaluating the efficacy of novel therapeutic agents, analyzing the process of tumor progression at the cellular and molecular level, and for the identification of new therapeutic targets. However, their utility depends heavily on preservation and stability of biological and morphological characteristics of the primary tumors. Confirmation of this stability is crucial in order to reliably identify molecular responses to treatment in xenografts which can be extrapolated back to patients.

In this report, we describe the establishment of primary mesothelioma cultures and patient derived tumor xenografts with mutational alterations that recapitulate those in the original patient tumors. We isolated malignant mesothelioma cells from the ascites or pleural effusion of five mesothelioma patients and grew them successfully in culture. Furthermore, we performed detailed morphologic and molecular characterization of early passage cultures of these cells and patient derived tumor xenografts in nude mice and assessed their malignant potential *in vitro*. Using a number of techniques, we demonstrated that these cells were of mesothelial origin and are indeed malignant. Four of the cell lines had *BAP1* mutations. We also showed a strong correlation between the mutation and the absence of expression of BAP1 protein. Three out of five cells, all of which were derived from *BAP1* mutant primary tumors, exhibited anchorage independent growth and also formed tumors *in vivo*, suggesting that BAP1 loss may enhance tumor growth *in vivo*. Both early passage cell cultures and mouse xenograft tumors harbored the *BAP1* mutations and *CDKN2A* deletions identical to those observed in the corresponding primary tumor. Given the similarities to the primary tumor, these models offer an opportunity in mesothelioma to study efficacy of novel therapeutic agents and to identify molecular responses to treatment. We believe these models would also enable further studies into functional aspects of *BAP1*, which is mutated in nearly a quarter of all mesotheliomas.

We also found a strong correlation between the ability of these cells to grow in an anchorage independent fashion *in vitro* and to induce tumors *in vivo*, similar to other work [6]. These features, along with the stable nature of these cell cultures, suggest that these early passage cells would be useful for functional and preclinical studies. It is noteworthy that the cell cultures that formed colonies *in vitro* and tumors in nude mice were cell lines that had *BAP1* mutations. Previous studies of BAP1 have shown that BAP1 loss promotes colony-forming ability of mesothelioma cells, and that re-expression of BAP1 in BAP1-deficient markedly decreases colony-formation [11]. Combined with the fact that these same cell cultures were also tumorigenic suggests that BAP1 loss may also enhance tumor growth *in vivo*.

Some *BAP1* missense mutations have been shown to affect the ubiquitin hydrolase activity of its protein product; however, splice site mutations cause exon-skipping leading to aberrant, out-of-frame transcripts [11]. A recent study has suggested that BAP1 inactivation is more closely associated with the epithelioid subtype of malignant pleural mesothelioma [28]. Consistent with this possibility, 3 of the 4 cell cultures harboring *BAP1* mutations in our study had epithelioid histology. Since the p16^INK4a^/p14^ARF^ proteins encoded by the *CDKN2A* locus are essential for normal cell cycle control, FISH analysis of this locus can be useful for the diagnosis of early-stage mesotheliomas of epithelial type [29]. Our finding of homozygous *CDKN2A* deletions in four of five cultures helps confirm the malignant nature of these cells. Recent experimental work has documented the importance of this locus to mesothelioma carcinogenesis. These studies have shown that mice deficient for either p16^Ink4a^ or p19^Arf^, the murine homolog of human p14^ARF^, have increased susceptibility to asbestos-induced mesothelioma and that inactivation of both p16^Ink4a^ or p19^Arf^ cooperate to accelerate asbestos-induced tumorigenesis [30].

Karyotypic analysis revealed recurrent abnormalities in the short arm of chromosome 1, consistent with a deletion hotspot previously implicated in mesothelioma [31,32]. Deletions of chromosome arms 3p, 9p and 22q, which include the tumor suppressor genes *BAP1*, *CDKN2A*, and *NF2*, respectively, have all been linked to mesothelioma. We also identified variable rearrangements of chromosome 10 in all our primary cultures, a finding that to our knowledge has not been reported earlier for malignant mesothelioma. Thus, investigations of the functional significance of these genomic hotspots, along with the mutational studies, may lead to the identification of molecular targets for the treatment of this disease.

## Conclusions

In summary, we describe the establishment of primary mesothelioma cultures and patient derived tumor xenografts with features that mirror the primary tumors. The primary cell cultures derived from ascitic or pleural fluids of patients with mesothelioma are highly tumorigenic and maintain the histologic and molecular features of the original tumors when grown in murine models. To our knowledge this is the first report that compares the characteristics of the patient’s tumors with the primary cells and mouse xenograft at the genetic level in malignant mesothelioma. Given that these primary cultures and patient derived tumor xenograft models recapitulate phenotypic and genetic features of the original primary mesotheliomas, they should prove useful for preclinical studies of novel drug regimens and for functional studies of *BAP1* biology in mesothelioma.

## Additional files

Additional file 1:
**Short tandem repeat analysis of 5 mesothelioma primary cells.**

Additional file 2: **Flow cytometry showing cell surface expression of mesothelin in early and late passage cells.** Cells were incubated with the anti-mesothelin mAb MN, conjugated with R-PE or isotype control antibody. Results are shown in terms of histogram plots for each cell line where the area under the blue line depicts the binding of MN antibody and the area under the red line shows the binding of isotype control antibody.
Additional file 3: Table S2. Karyotyping of early and late passage primary mesothelioma cell lines.
Additional file 4:
**Western blot images showing the expression E-cadherin, N-cadherin and vimentin in primary cell cultures.**

## Abbreviations

COLD: Co-amplification at lower denaturation temperature
FISH: Fluorescence in situ hybridization
IHC: Immunohistochemistry
PBS: Phosphate buffered saline
PCR: Polymerase Chain Reaction
STR: Short Tandem Repeats
SNVs: Single-nucleotide variants
