# Supplementary material for: Mesothelioma patient derived tumor xenografts with defined BAP1 mutations that mimic the molecular characteristics of human malignant mesothelioma
Source: BMC Cancer. 2015 May 8;15:376. doi: 10.1186/s12885-015-1362-2 (PMC4431029; doi:10.1186/s12885-015-1362-2)
Supplement: Additional file 1: — Short tandem repeat analysis of 5 mesothelioma primary cells. [file 12885_2015_1362_MOESM1_ESM.doc]

| **Additional file 1**: Short tandem repeat analysis of 5 mesothelioma primary cells and passage 3 and passage 30 | | | | | | | | | | | | |
| --- | --- | --- | --- | --- | --- | --- | --- | --- | --- | --- | --- | --- |
|  | **NCI-Meso16**  **P3 P30** | | **NCI-Meso17**  **P3 P30** | | **NCI-Meso18**  **P3 P30** | | **NCI-Meso19**  **P3 P30** | | **NCI-Meso21**  **P3 P30** | | **Test positive control** | **Expected Positive control** |
|  | P3 | P30 | P3 | P30 | P3 | P30 | P3 | P30 | P3 | P30 |  |  |
| **D8S1179** | 14,15 | 14,15 | 12 | 12 | 14,15 | 14,15 | 12,15 | 12,15 | 12,14 | 12,14 | 13 | 13 |
| **D21511** | 29, 31.2 | 29, 31.2 | 28,29 | 28,29 | 30 | 30 | 32.2 | 32.2 | 29 | 29 | 30 | 30 |
| **D75820** | 12, 14 | 12, 14 | 10,12 | 10,12 | 10,12 | 10,12 | 9 | 9 | 9,11 | 9,11 | 10,11 | 10,11 |
| **CSF1P0** | 11 | 11 | 11 | 11 | 10 | 10 | 8,10 | 8,10 | 10 | 10 | 10,12 | 10,12 |
| **D351358** | 18 | 18 | 18 | 18 | 15 | 15 | 15 | 15 | 14 | 14 | 14,15 | 14,15 |
| **TH01** | **9,9.3** | **9.3** | 8,9 | 8,9 | 6 | 6 | 7,9 | 7,9 | 9.3 | 9.3 | 8,9.3 | 8,9.3 |
| **D135317** | 11 | 11 | 13 | 13 | 8,11 | 8,11 | 12 | 12 | 11,13 | 11,13 | 11 | 11 |
| **D165539** | 11,13 | 11,13 | 11 | 11 | 8 | 8 | 12 | 12 | **9** | **9,10** | 11,12 | 11,12 |
| **D251338** | 17,24 | 17,24 | 17 | 17 | 23,25 | 23,25 | 25 | 25 | 17,26 | 17,26 | 19,23 | 19,23 |
| **D195433** | 13 | 13 | 13,15 | 13,15 | **15.2** | **14,15.2** | 12,14 | 12,14 | 10,16 | 10,16 | 14,15 | 14,15 |
| **VWA** | 16,17 | 16,17 | 16,18 | 16,18 | 15,18 | 15,18 | 15,19 | 15,19 | 15,18 | 15,18 | 17,18 | 17,18 |
| **TPOX** | 8,11 | 8,11 | 9 | 9 | 8 | 8 | 9 | 9 | 8,11 | 8,11 | 8 | 8 |
| **D18S51** | 18 | 18 | 15 | 15 | **12** | **12,13** | 18 | 18 | 12,16 | 12,16 | 15,19 | 15,19 |
| **D55818** | 11,12 | 11,12 | 11,12 | 11,12 | 11 | 11 | 11,13 | 11,13 | 11,12 | 11,12 | 11 | 11 |
| **FGA** | **19** | **19,25** | 24 | 24 | **20** | **20,23** | **20,22** | **20** | 20,23 | 20,23 | 23,24 | 23,24 |
| **Amelogenin** | XY | XY | X | X | XY | XY | X | X | XY | XY | X | X |
