# Supplementary figures and images for: Mesothelioma patient derived tumor xenografts with defined BAP1 mutations that mimic the molecular characteristics of human malignant mesothelioma
Source: BMC Cancer. 2015 May 8;15:376. doi: 10.1186/s12885-015-1362-2 (PMC4431029; doi:10.1186/s12885-015-1362-2)

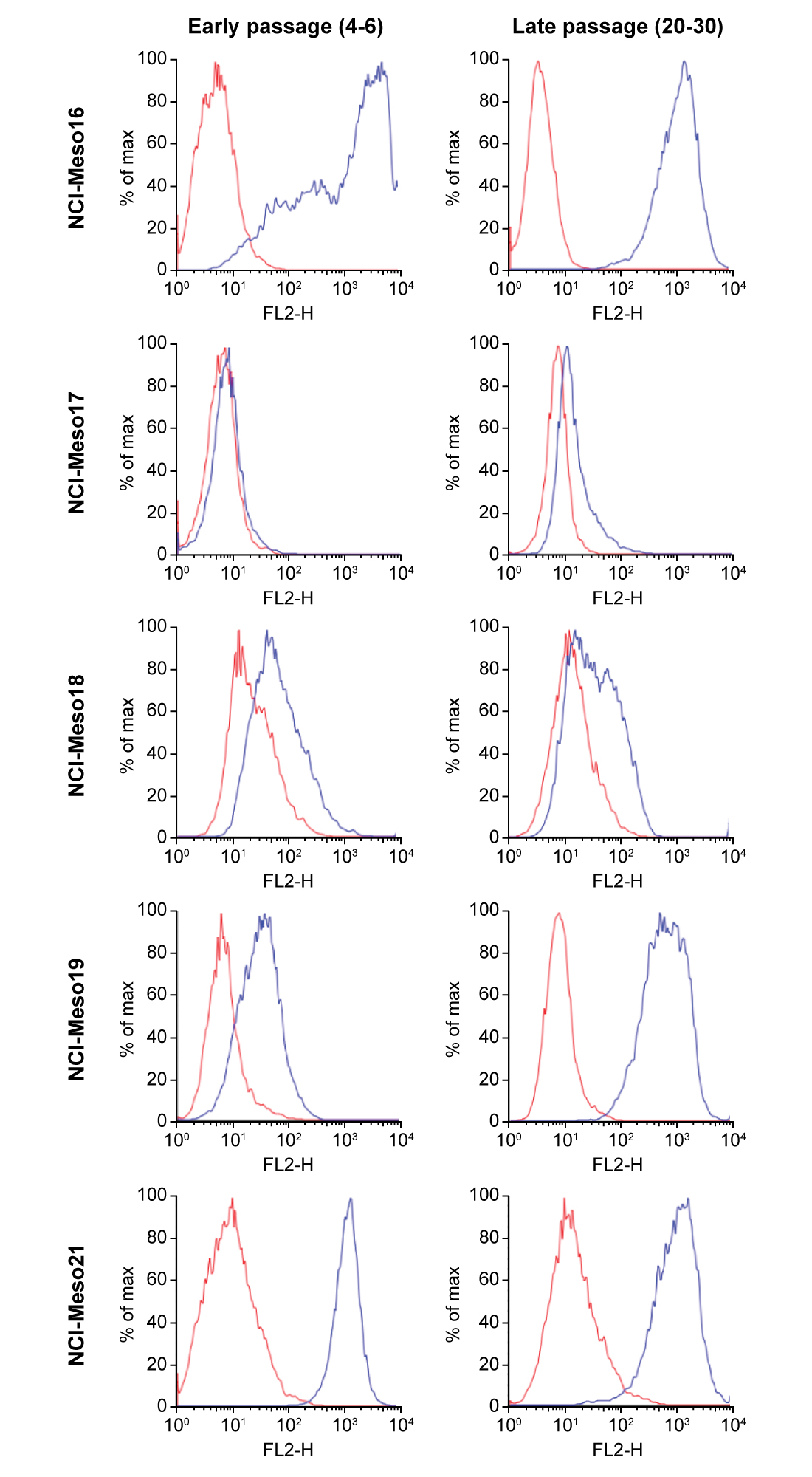

Supplement: Additional file 2: — Flow cytometry showing cell surface expression of mesothelin in early and late passage cells. Cells were incubated with the anti-mesothelin mAb MN, conjugated with R-PE or isotype control antibody. Results are shown in terms of histogram plots for each cell line where the area under the blue line depicts the binding of MN antibody and the area under the red line shows the binding of isotype control antibody. [file 12885_2015_1362_MOESM2_ESM.jpeg]

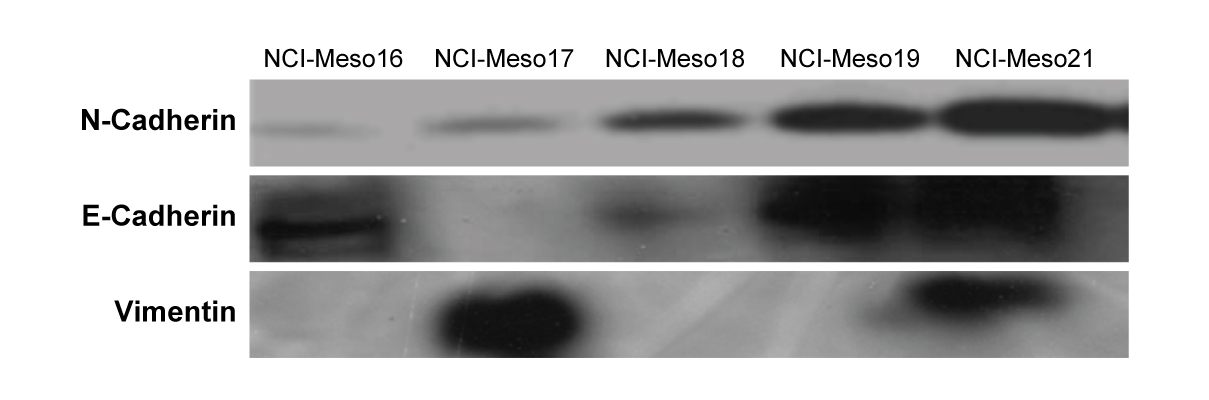

Supplement: Additional file 4: — Western blot images showing the expression E-cadherin, N-cadherin and vimentin in primary cell cultures. [file 12885_2015_1362_MOESM4_ESM.jpeg]
