# Supplementary material for: Mesothelioma patient derived tumor xenografts with defined BAP1 mutations that mimic the molecular characteristics of human malignant mesothelioma
Source: BMC Cancer. 2015 May 8;15:376. doi: 10.1186/s12885-015-1362-2 (PMC4431029; doi:10.1186/s12885-015-1362-2)
Supplement: Additional file 3: Table S2. — Karyotyping of early and late passage primary mesothelioma cell lines. [file 12885_2015_1362_MOESM3_ESM.doc]

| **Additional file 3**: Karyotyping of early and late passage primary mesothelioma cell lines | | | |
| --- | --- | --- | --- |
| Cell Line | Early passage (passage 3) | Late passage (passages 27-31) | Changes noted at high passage |
| NCI-Meso16 | 60,XXYY,del(1)(q42;q44),+der(1)t(19;1)(p12;q10),+2,+del(3)(p26;p14),+5,+10,+del(11)(p15),+12,+12,+der(15)t(15;1;15),+16,+17,+der(20)t(20;15) | 57,XXYY,del(1)(q42;q44),+der(1)t(19;1)(p12;q10),+der(3)t(2;3),+5,+10,+del(11)(p15),+12,+16,+17,+der(20)t(20;15) | Loss of addition 2, loss of deletion 2  Change to t(2;3),loss of deletion 3  Single additional 12  Loss der(15)t(15;1;15) not seen |
| NCI-Meso17 | 47,X,-X,  der(2)t(2;10)(q24~q31;q22~q24),  der(3)t(18;9;3),-4,+8,+8,der(9)t(9;15), i(13)(q10),der(18)t(18;9),del(20),+21 | 64,XX,-X,  +1,+2,der(2)t(2;10)(q24~q31;q22~q24),  +3,der(3)t(18;9;3),-4,+5,  -6,+7,+8,+8,+9,der(9)t(9;15),+10,-11,+12, i(13)(q10),-14,+15,+16,+17,+17,-18,der(18)t(18;9),+19,+del(20)+20,+21,+21,-22 | Rearrangements remained the same  Transition to near trisomy |
| NCI-Meso18 | 72,X,t(X;5),der(Y)t(Y;6;Y),der(Y),?t(10;Y;6),  -1,t(1;3)x2,+t(1;18),t(22;2),+t(22;2),del(3)x2,  t(4;20),+t(4;1),+t(2;5), i(6),t(6;21), +7,+del(7),i(8)q(10), t(4;8),+t(18;8)x2, del(9), t(21;9;1),+t(13;9), t(2:10),+t(7;10),+(Y;10),+del(11),+t(3;11)+12,+12,+t(13;9;21),t(14;6)x2,+15,+t(15;20),+16,+16, +17, -18, +del(19), +der(21) | 72,X,t(X;5),der(Y)t(6:Y),der(Y)?t(10;Y), t(1;3)x2,+t(1;18)x2, +del(1)+t(22;2)x2, del(3)x2),t(4;20), +del(4),+del(5),+t(5;X), +i(6), +7,+del(7), +i(8), del(9),t(9;19),t(13;9)x2, +t(2;10),+t(10;Y), del(11),+t(3;11),+12, t(14;6)x2, +15, +t(15;20),+16,+t(4;16),-18,+del(19),del(19),t(21;20),der(21), add(22),+22 | Apparent reciprocal t(X;5) in late passage  Early passage contained t(4;1),t(2;5)  Late passage contain a del(1), t(4;16) and t(21;20) |
| NCI-Meso19 | 39,der(X)t(X;22,14),Y,der(1)t(1q;7q),  der(1)t(1;12),der(3)t(3;8),-3,t(4;3),  del(4q),der(5)t(5;Y),-6,der(6)t(6;2),-7, del(7)?t(22;7),der(7)t(7p;1p),-8 der(9)t(7;9;3),del(9),t(6;10),der(11)t(3;11),  t(12;22)-13,t(6;13),-14x2,del(15)+t(4;15),  -18, der(18)t(18;3), 21,-22 | 38,der(X)t(X;22;14),Y,der(1)t(1q;7q),  der(1)t(1;12),der(3)t(3;8),-3,t(4;3),  del(4q),der(5)t(Y;5),- 6, der(6)t(6;2),-7, del(7),der(7)t(7p;1p),-8,der(9)t(7;9;3),  del(9),der(10)t(6;10),der(11)t(3;11),-12 -13,t(6;13),-14x2,del(15),+t(4;15),-18, der(18)t(18;3), 21,-22 | None |
| NCI-Meso21 | 44,XY,del(1)(p36.5-p21),+3,der(6)t(6;21),  der(7)t(1;7;15),der(9)t(10;9), der(10)t(2;10;7),t(10;19),del(15)(~q22), t(19;10),-21,-22 | 45,XY,del(1)(p36.5-p21),+3,der(6)t(6;21),  der(7)t(1;7;15),der(9)t(10;9), der(10)t(2;10;7),t(10;19),t(14;1), del(15(~q22),t(19;10),-21,-22 | Of late passage cells, 3 in 10 metaphases carried t(14;1) |
